# Global transcriptomics identification and analysis of transcriptional factors in different tissues of the paper mulberry

**DOI:** 10.1186/s12870-014-0194-6

**Published:** 2014-08-20

**Authors:** Xianjun Peng, Yucheng Wang, Ruiping He, Meiling Zhao, Shihua Shen

**Affiliations:** Key Laboratory of Plant Resources, Institute of Botany, The Chinese Academy of Sciences, 100093 Beijing, People’s Republic of China; University of the Chinese Academy of Sciences, 100093 Beijing, People’s Republic of China

**Keywords:** Lateral organ development, Paper mulberry, Phylogeny, Root hair elongation, Transcription factors

## Abstract

**Background:**

The paper mulberry (*Broussonetia papyifera*) is one of the multifunctional tree species in agroforestry system and is also commonly utilized in traditional medicine in China and other Asian countries. To identify the transcription factors (TFs) and comprehensively understand their regulatory roles in the growth of the paper mulberry, a global transcriptomics TF prediction and the differential expression analysis among root, shoot and leaf were performed by using RNA-seq.

**Results:**

Results indicate that there is 1,337 TFs encoded by the paper mulberry and they belong to the 55 well-characterized TF families. Based on the phylogenetic analysis, the TFs exist extensively in all organisms are more conservative than those exclusively exist in plant and the paper mulberry has the closest relationship with the mulberry. According to the results of differential expression analysis, there are 627 TFs which exhibit the differential expression profiles in root, shoot and leaf. *ARR-B*s, *ARF*s, *NAC*s and *bHLH*s together with other root-specific and highly expressed TFs might account for the developed lateral root and unconspicuous taproot in the paper mulberry. Meanwhile, five *TCP*s highly expressed in shoot of the paper mulberry might negatively regulate the expression of 12 *LBD*s in shoot. Besides, LBDs, which could directly or indirectly cooperate with ARFs, bHLHs and NACs, seem to be the center knot involving in the regulation of the shoot development in the paper mulberry.

**Conclusions:**

Our study provides the comprehensive transcriptomics identification of TFs in the paper mulberry without genome reference. A large number of lateral organ growth regulation related TFs exhibiting the tissue differential expression may entitle the paper mulberry the developed lateral roots, more branches and rapid growth. It will increase our knowledge of the structure and composition of TFs in tree plant and it will substantially contribute to the improving of this tree.

**Electronic supplementary material:**

The online version of this article (doi:10.1186/s12870-014-0194-6) contains supplementary material, which is available to authorized users.

## Background

The paper mulberry belongs to the family of Moraceae and is naturally distributed in Eastern Asia and pacific countries. The paper mulberry has shallow roots with advanced lateral roots and without an obvious taproot. It is one of the multifunctional tree species in agroforestry systems [1], as well as being one of the traditional forages [2] and Chinese medicines in many countries of Asia [3]. Due to its fast growth and adaptability, the paper mulberry is commonly used for the ecological afforestation and landscape in both sides of highway, mined areas and on barren land [4]. It is the ideal tree species for ecological and gardening purposes, and can be widely used in papermaking, livestock, medicine and other industries [5]. Genetic diversity revealed by SRAP marker and cluster analysis show that there is a relationship between the genetic variation and geographical distribution [6]. These results provide reference for making genetic map and guide the breeding of the paper mulberry. However, because of lacking the knowledge of the genetic background, the molecular mechanism about strong adaptability and tolerance to biotic or abiotic stress of the paper mulberry has not been studied, which limits the exploitation of the paper mulberry.

TFs play important roles in plant development and environmental adaptation by regulating the expression of their target genes. TFs directly or indirectly involved in the response to plant hormones which control plant growth including cell division, elongation and differentiation. The identification and functional study of TFs are essential for the reconstruction of the transcriptional regulatory network in the development and ecological circumstances adaptation of plant. Many TF family proteins, such as bHLH [7], ERF [8], Dof [9], MYB [10], NAC [11] and WRKY [12], play regulatory roles in plants growth and development.

Many TFs have been reported to play roles in the vascular and xylem development [13]. Recent molecular studies of various trees have revealed that the coordinated gene expression during differentiation of these cells in wood and the presence of several TFs, such as ARF, HD-ZIP, MYB and NAC, which might govern the complex networks of transcriptional regulation in tree growth [14]. However, most studies about genome wide analysis of TFs in plants concentrate in a few species, such as Arabidopsis and kinds of crops. The universality of the mechanism is not explicit, especially in tree species. Because of low domestication, open-pollinated native populations and high levels of genetic variation, they are ideal organisms to unveil the molecular mechanism of population adaptive divergence in nature.

As nonclassical model plant, trees have gained much attention in recent years for environment adaptation, evolutionary and genomic studies. Overall study for each TF family has also been launched. Via the comprehensive analysis of NAC gene family in *Populus*, a total of 163 full-length NAC genes are identified, and they are phylogenetically clustered into 18 distinct subfamilies. Furthermore, 25 NAC genes are of tissue-specific expression patterns [15]. A total of 11 *WOX* TFs both mRNA and genomic DNA are isolated from *Picea abies* and further study shows that all major radiations within the WOX gene family taking place before the angiosperm-gymnosperm split and that there has been a recent expansion within the intermediate clade in the Pinaceae family [16]. However, there are little reports about the regulated network from the genome-scale under the control of TFs in tree species [13,17], especially as for those trees without genome information.

In our study, we performed a genome wide TF prediction using the transcriptome data. Additionally, we predicted the expressional pattern of the identified TF genes using a large amount of RNA-seq data which have just become available. A subset of TFs that are specifically expressed in particular tissues, including root, shoot and leaf, were thus identified. Our study provides a valuable resource of TF genes for further genetic and developmental studies in the paper mulberry.

## Results

### Identification and classification of TFs in the paper mulberry

To ascertain the TF families in the paper mulberry, sequences obtained from 3 libraries as mentioned in the materials and methods were assembled. After retrieving annotation results for every unigene, 1,337 TFs were identified and classified into 55 families (Table 1) based on their DNA-binding domains and other conserved motifs [18,19]. Of these TFs, 578 TFs belonged to 48 families with complete ORF (Table 2). The bHLH was the biggest family with 151 members and 69 of which have complete ORF. The following was WRKY (112), ERF (88) and other families. According to comparison of family size among the selected species, as shown in Table 2, most of families have been detected in the paper mulberry except for GeBP, HB-PHD, MIKC, and STAT families.

**Table 1:** **TF family in paper mulberry**

**TF family**	**No. of TF**	**TF family**	**No. of TF**	**TF family**	**No. of TF**	**TF family**	**No. of TF**
Alfin-like	16	CPP	8	HTH	13	S1Fa-like	1
AP2	25	DBB	5	LBD	26	SAP	2
ARF	26	Dof	28	LFY	2	SBP	21
ARR-B	33	E2F/DP	9	LSD	1	SRS	1
B3	53	EIL	4	M-type	13	TALE	19
BBR-BPC	5	ERF	88	MYB	83	TCP	18
BES1	6	FAR1	50	MYB-related	33	Trihelix	13
bHLH	151	G2-like	29	NAC	79	VOZ	2
BTF3	1	GATA	24	NF-X1	2	Whirly	2
bZIP	35	GRAS	29	NF-YA	6	WOX	6
C2H2	64	GRF	16	NF-YB	5	WRKY	112
C3H	67	HD-ZIP	35	NF-YC	3	YABBY	3
CAMTA	4	HRT	1	Nin-like	6	ZF-HD	11
CO-like	19	HSF	21	RAV	2

**Table 2:** **TF families with complete ORF in paper mulberry**

**TF family**	**No. of TF**	**TF family**	**No. of TF**	**TF family**	**No. of TF**	**TF family**	**No. of TF**
Alfin-like	8	CO-like	9	GRF	4	RAV	1
AP2	8	CPP	3	HD-ZIP	21	SBP	11
ARF	12	DBB	1	HSF	13	SRS	1
ARR-B	14	Dof	9	LBD	9	TALE	7
B3	17	E2F/DP	6	MADS-box	4	TCP	8
BBR-BPC	4	EIL	3	MYB	30	Trihelix	8
bHLH	69	ERF	44	MYB-related	9	VOZ	2
BTF3	1	FAMILY	1	NAC	21	Whirly	2
bzip	24	FAR1	24	NF-X1	2	WOX	3
C2H2	19	G2-like	21	NF-YA	3	WRKY	33
C3H	41	GATA	14	NF-YB	4	YABBY	3
CAMTA	4	GRAS	19	NF-YC	1	ZF-HD	3

### Phylogenetic analysis of TFs in the paper mulberry

**Figure 1:**
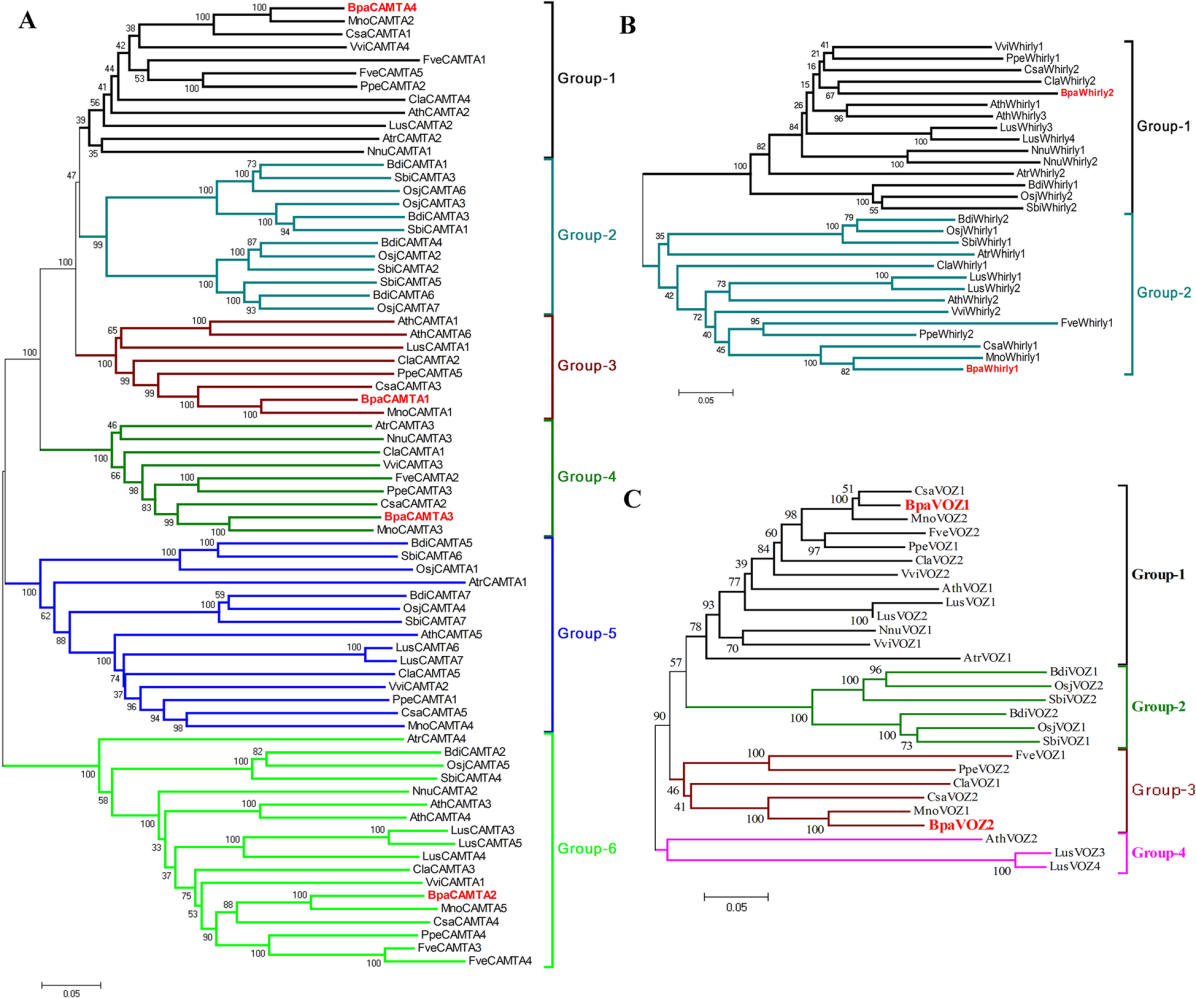
**Evolutionary relationships revealed by phylogenetic analysis of CAMTA, Whirly and VOZ family.** The evolutionary history was inferred using the Neighbor-Joining method. The percentage of replicate trees in which the associated taxa clustered together in the bootstrap test (1500 replicates) was shown next to the branches. The tree was drawn to scale, with branch lengths in the same units as those of the evolutionary distances used to infer the phylogenetic tree. The evolutionary distances were computed using the p-distance method and were in the units of the number of amino acid differences per site. The analysis involved 74 CAMTAs **(A)**, 29 Whirlys **(B)** and 27 VOZs **(C)** amino acid sequences and their correspondent accession numbers were list in Additional file 4: Table S6. All ambiguous positions were removed for each sequence pair. Evolutionary analyses were conducted in MEGA5.

Genetic distances were calculated according to the alignment of the conserved domain of the three TFs families chosen from 9 species including the paper mulberry and the phylogenetic trees were constructed using MEGA 5.0 program (Figure 1). As shown in Figure 1A, all of the CAMTAs from the selected species could be classified into six groups. Four BpaCAMTAs were listed in the group 1, 3, 4 and 6, respectively. All of the BpaCAMTAs were clustered with that from *Morus notabilis*, following as *Cannabis sativa*. There were two Whirly TFs in the paper mulberry and they were divided into two groups as that of other plants. One of them was clustered with that of *M. notabilis* and the other was clustered with *Citrullus lanatus* (Figure 1B). Two VOZs existed in the paper mulberry like as most of other plants and they had the highest identity with that of *M. notabilis* and *C. sativa* (Figure 1C).

### The expression profile of the TFs from the paper mulberry

To identify the differentially expressed TFs between different samples, the expression level of all TFs were homogenized by using their RPKM values. Among the 1,337 TFs, the RPKM values of 1,104 TFs were distributed from 1 to 770 (see Additional file 1: Table S4). The unigene T6-23630 had the highest RPKM value 770 and belonged to the ERF family. Besides, there were 219 TFs which RPKM values were approximate to zero and belonged to the bHLH, WRKY and other families. They had a common characteristic of the short nucleotide length which distributed from 202 to 309 bp.

**Figure 2:**
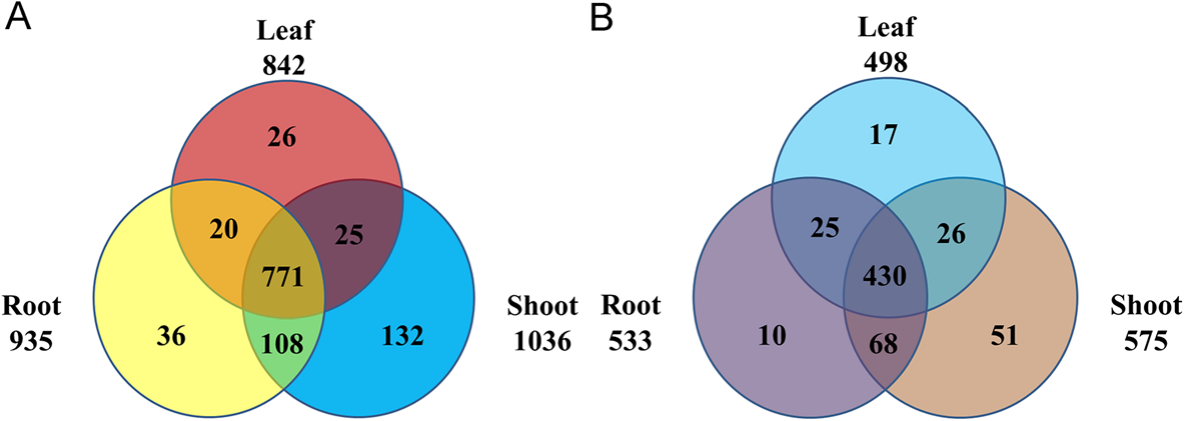
**Venn diagram of TFs expressed and differently expressed in root, shoot and leaf. A** Venn diagram of TFs expressed in root, shoot and leaf. **B** Venn diagram of differentially expressed TFs in root, shoot and leaf. TFs differentially expressed in root, shoot and leaf were identified. To be considered differentially expressed, the transcript must have RPKM ≥ 2 in at least one tissue, 2-fold or greater change between tissues, and P ≤ 0.05.

According to the RPKM value of each TF, there were 935, 1036 and 842 TFs expressed in the root, shoot and leaf, respectively (Figure 2A). A total of 771 TFs were co-expressed in three tissues. Meanwhile, there were 36, 132 and 26 TFs were specifically expressed in root, shoot and leaf, respectively.

### Differentially expressed TFs from the paper mulberry

**Figure 3:**
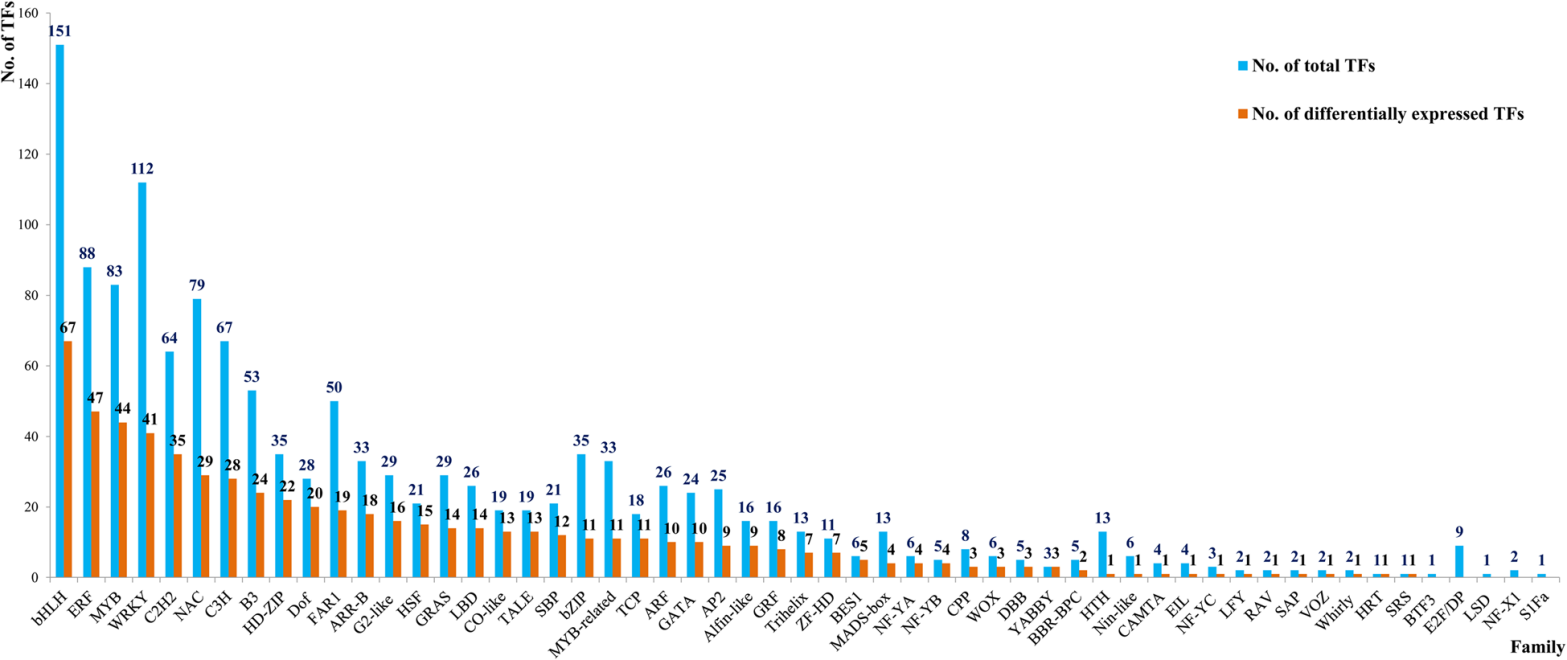
**The differentially expressed TFs distributed in each family.** According to the conserved domain, a total of 627 differentially expressed TFs could be classified into 50 families and most of them concentrated in bHLH, MYB, WRKY, C2H2, NAC, C3H, B3 and Dof family.

**Figure 4:**
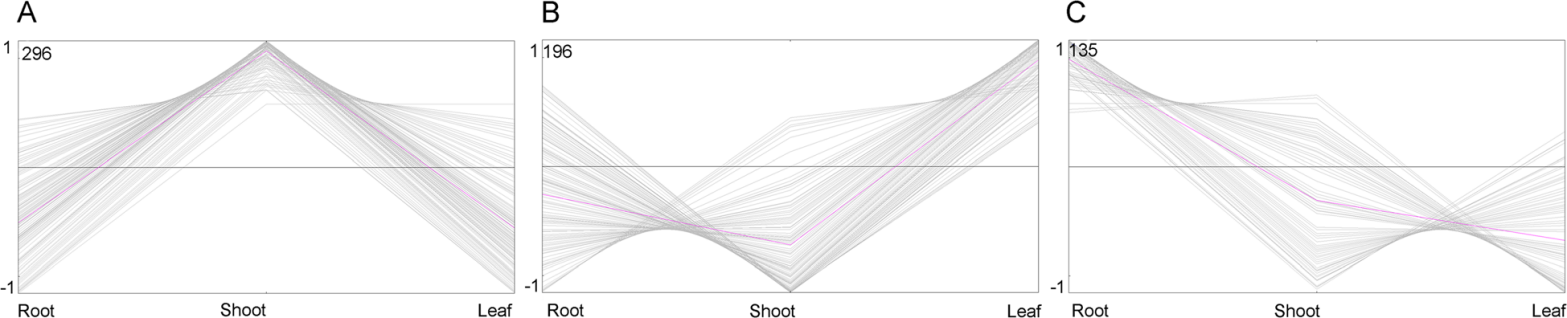
**The cluster analysis of the differentially expressed TFs in root, shoot and leaf of the paper mulberry. A** The TFs highly expressed in shoot than that in leaf and root. **B** The TFs highly expressed in leaf than that in shoot and root. **C** The TFs highly expressed in root than that in leaf and shoot. The pink line represented the expression trend of the cluster. The gray line represented the expression profile of every TF.

**Figure 5:**
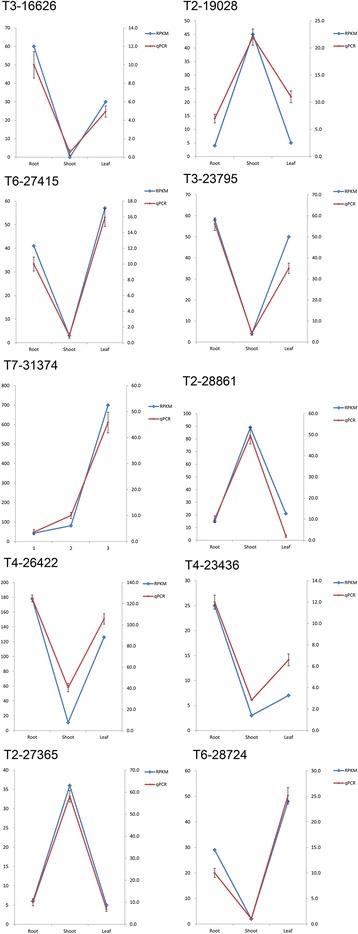
**The expression profile of ten selected TFs validated by qPCR.** The left axis represents the results of transcriptomics analysis while the right axis represents relative expression detected by qPCR, and the error bars represented the standard deviation (S.D.) values for three independent experiments, performed in triplicate.

The TFs with a RPKM value of more than or equal to 2 were chosen for the differential expressed analysis and The TFs with a ratio of RPKM between samples of more than 2 (Fold change ≥2) and an FDR ≤0.01 were considered to have the significant changes in expression. According to this rule, a total of 627 TFs were of the differential expressed characteristic among root, shoot and leaf (see Additional file 2: Figure S1 and Additional file 3: Table S5, Figure 2B) and belonged to AP2, CO-like, LBD and other 47 families (Figure 3 and see Additional file 2: Figure S1). There were 135, 296 and 196 TFs had the highest expression level in root, shoot and leaf, respectively (Figure 4A, B and C). Among of them, there were 10, 51 and 17 TFs were uniquely expressed in the root, shoot and leaf, respectively (Figure 2B). These expression patterns were validated by qPCR (Figure 5) and the error bars showed the corresponding standard deviation when three independent experiments were carried out. In addition, there were 332 TFs, belonged to 42 families, had complete ORFs among these tissue differential expressed TFs (see Additional file 4: Table S6).

## Discussion

### The composition of TFs in the paper mulberry

**Figure 6:**
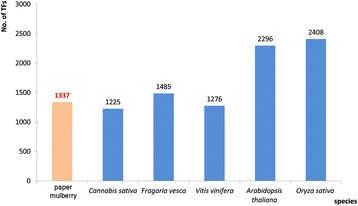
**The total number of TFs in the selected species.** The TF numbers of *Arabidopsis thaliana*, *Cannabis sativa*, *Fragaria vesca*, *Oryza sativa* subsp. *Japonica* and *Vitis vinifera* were obtained from Plant Transcription Factor Database (http://planttfdb.cbi.pku.edu.cn/index.php).

TFs are usually classified into different families based on their DNA-binding domains and other conserved motifs [18,19]. As the model plants of dicots and monocots, the genomes of Arabidopsis and rice have been well discerned. The Arabidopsis genome encodes 2,296 TFs which can be classified into 58 families and account for 6.2% of its estimated total number of genes [18,20]. There are 2,408 TFs (1,859 loci) are identified and classified into 56 families in *Oryza sativa subsp. Japonica*. Furthermore, there are 4,288 TFs encoded by 2,455 genes accounting for about 6.4% of Poplar gene [18,21]. In our study, a total of 1,337 TFs identified from the transcriptome data of the paper mulberry could be classified into 55 families and 578 TFs of them had complete ORF. These TFs comprised of more than 3.5% of the genes of this plant [22]. Although the genome of paper mulberry has not been sequenced and its genes number might be underestimated, this ratio was much closed to that of other genome known plants, such as *C. sativa*, *Fragaria vesca* and *Vitis vinifera* (Figure 6) and it is less than that of Arabidopsis and rice. Besides, the TFs number of bHLH, AP2/ERF, MYB/MYB-related, NAC and WRKY family in the paper mulberry was 151, 114, 116, 79 and 112, respectively. They mostly made up half of the total TFs of the paper mulberry just as other plants (Table 3).

**Table 3:** **The comparison of family size among the selected species**

**TF family**	***Oryza sativa***	***Arabidopsis thaliana***	***Vitis vinifera***	***Fragaria vesca***	***Cannabis sativa***	**Paper mulberry**	**TF family**	***Oryza sativa***	***Arabidopsis thaliana***	***Vitis vinifera***	***Fragaria vesca***	***Cannabis sativa***	**Paper mulberry**
AP2	22	30	19	17	16	25	LBD	39	50	44	36	26	26
ARF	48	37	17	17	13	26	LFY	2	1	1	4	1	2
ARR-B	11	21	12	7	5	33	LSD	12	12	3	3	3	1
B3	65	77	29	77	60	53	MIKC	61	76	36	37	28	-
BBR-BPC	7	17	5	3	3	5	M-type	35	70	18	48	6	13
BES1	6	14	6	6	8	6	MYB	130	168	138	110	81	83
bHLH	211	225	115	112	99	151	MYB-related	106	97	57	65	70	33
bZIP	140	127	47	52	54	35	NAC	170	138	71	127	75	79
C2H2	135	116	64	83	62	64	NF-X1	2	2	3	2	4	2
C3H	74	66	43	38	48	67	NF-YA	25	21	7	6	6	6
CAMTA	7	10	4	7	6	4	NF-YB	16	27	17	14	12	5
CO-like	21	22	6	7	10	19	NF-YC	19	21	8	9	8	3
CPP	20	9	6	6	3	8	Nin-like	15	17	8	10	4	6
DBB	13	14	7	6	5	5	NZZ/SPL	-	1	1	1		-
Dof	37	47	22	23	27	28	RAV	4	7	1	5	2	2
E2F/DP	10	16	7	7	11	9	S1Fa-like	2	4	2	1	1	1
EIL	11	6	2	6	5	4	SAP	-	1	1	1		2
ERF	163	139	80	92	59	88	SBP	29	30	19	14	18	21
FAR1	133	26	18	82	36	50	SRS	6	16	5	5	7	1
G2-like	62	64	40	37	31	29	STAT	1	4	1	1	2	-
GATA	32	41	19	19	21	24	TALE	45	33	21	18	17	19
GeBP	13	23	1	6	5	-	TCP	23	33	15	18	17	18
GRAS	69	37	43	51	54	29	Trihelix	40	34	26	31	33	13
GRF	19	9	8	10	10	16	VOZ	2	3	2	2	2	2
HB-other	17	11	7	11	14	-	Whirly	2	4	2	1	3	2
HB-PHD	1	3	2	2	1	-	WOX	17	18	11	14	7	6
HD-ZIP	61	58	33	28	34	35	WRKY	128	90	59	58	49	112
HRT-like	1	2	1	3	2	1	YABBY	15	8	7	5	7	3
HSF	38	25	19	15	26	21	ZF-HD	15	18	10	9	8	11

Note: “-” means no TF presented in this family.

However, GeBP, HB-PHD, MIKC, and STAT families were not found in our transcriptome data (Table 3). Meanwhile, the MIKC-type, specific to plants and involved in floral organ identity determination, and NZZ/SPL TFs, playing a central role in regulating anther cell differentiation during the floral organ development, were also not appeared in the transcriptome data. This might mainly because that the fruit and flower were not included in this study. There might some new TF members would be presented when more transcriptome data could be obtained.

### The phylogenetic relationship of the TFs in the paper mulberry

The mutation, expansion and functional diversification of gene family reflect the evolution process of plants to adapt to their differing external ecological circumstances. In our study, we chose three TF families, namely CAMTA, Whirly and VOZ, to illustrate the phylogenetic relationship of the TFs in the paper mulberry.

Investigations of CAMTAs in various organisms suggest a broad range of functions from sensory mechanisms to embryo development and growth control [23]. The CAMTAs have been shown to play an important role in the plant response to abiotic and biotic stresses [24]. Meanwhile, the CG-1, ANK and the IQ domain is very conservative from human to plant [23]. The phylogenetic tree of CAMTAs in our study showed that all of the BpaCAMTAs were clustered with that from *M. notabilis*, following was *C. sativa* (Figure 1A). The CAMTAs from *A. trichopoda* were located in the root position of group-1, 4, 5 and 6. This results was in accordance with that *A. trichopoda* was the single living species of the sister lineage and the most recent common ancestor to all other extant flowering plants [25]. In addition, the CAMTAs have evolved a novel clade in group 2 in *B. distachyon*, *O. sativa* and *S. bicolor* which confirmed that some gene family in monocots had rapidly evolved to adapt to the environment after the monocot-dicot divergence.

Whirly TFs throughout the plant kingdom are predicted to share the ability to bind to single-stranded DNA and they regulate defense gene expression as well as function in the chloroplast and in the nucleus [26]. Two Whirly TFs of the paper mulberry were divided into two groups as that of other plants (Figure 1B). One of them was clustered with that of *M. notabilis*. The other was clustered with that of *C. lanatus.* This suggested that the BpaWhirly1 was more conserved than BpaWhirly2.

VOZ is the plants specific one-zinc-finger type DNA-binding protein and is highly conserved in land plant evolution [27]. BpaVOZ1 was clustered with CsaVOZ1 while BpaVOZ2 was clustered with MnoVOZ1 (Figure 1C). This result showed that BpaVOZ1 had the higher identity with CsaVOZ1 other than MnoVOZ2 and implied that BpaVOZ1 and CsaVOZ1 have produced some similar mutation both in *C. sativa* and the paper mulberry.

*C. sativa* which has once been considered as one species of Moraceae in the Engler system [28] belongs to the Cannabaceae while *C. lanatus* belongs to the Cucurbitales (APG III Classification system). Even so, some TFs identified from the paper mulberry still had the higher identity with the TFs of them. These phylogenetic analyses suggested that the TFs existing in various organisms and playing the significant roles, such as CAMTAs, also were conserved in the paper mulberry. Meanwhile, the TFs which are specific to plants, for example VOZ and Whirly experienced a lower selection pressure, had more of the variation in the paper mulberry.

### TFs involved in the tissue growth of the paper mulberry

A TF that expresses exclusively in a special tissue may play a central role in regulating tissue development [29]. Expression patterns contain important information to infer the functions of TFs. Transcriptome-wide identification of tissue-specific TFs across tissues can help to understand of the molecular mechanisms of tissue development. The plantlet of the paper mulberry was in seedling stage with vigorously vegetative growth and without reproductive growth. So the key TFs involved in the regulation of root, shoot and leaf development could be identified by detecting the expression profile and screening the tissue-specific expression.

#### Root growth

**Figure 7:**
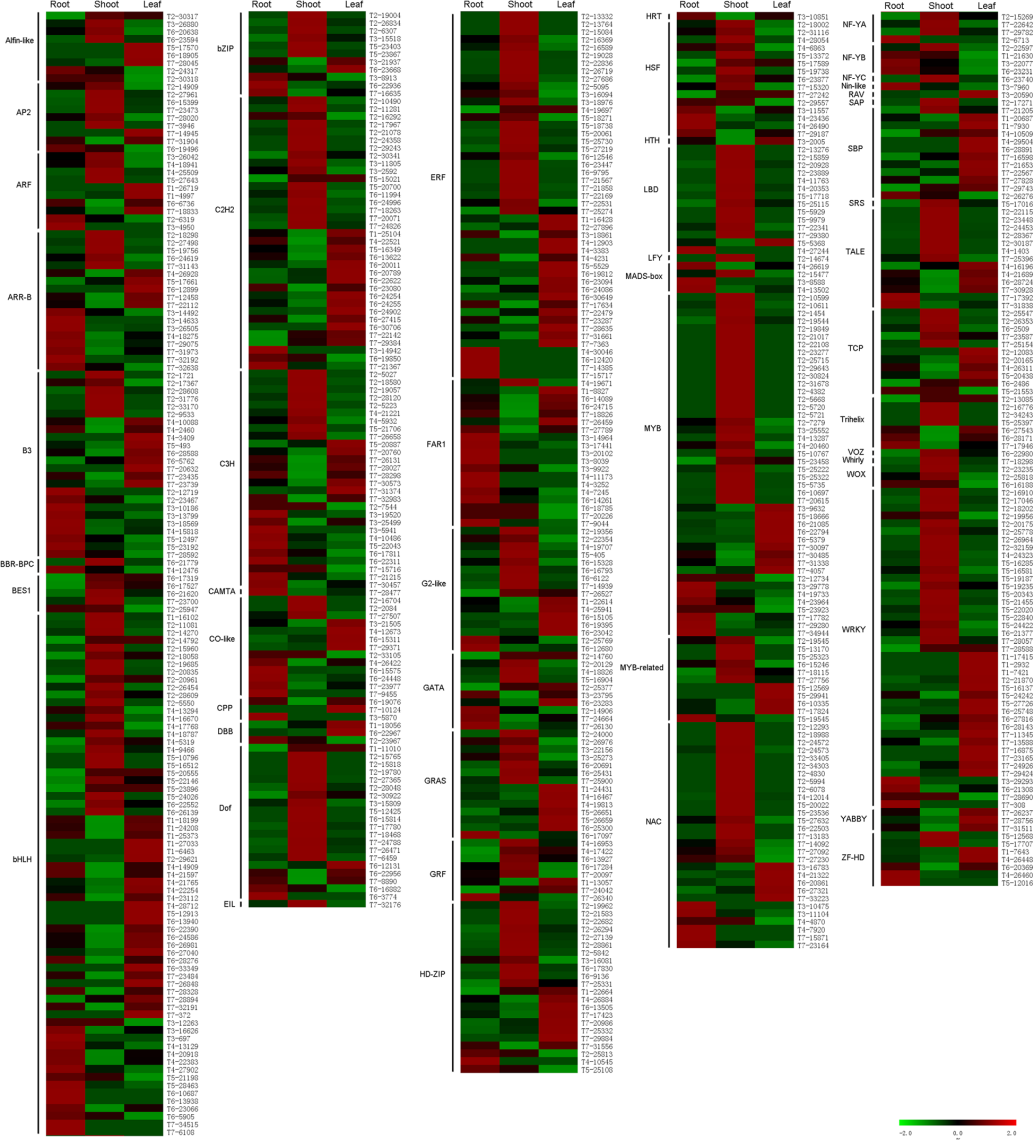
**Heat map of expression profiles of TFs involved in the differential expression among root, shoot and leaf.** Red indicates high expression, black indicates intermediate expression, and green indicates low expression. To be considered differentially expressed, the transcript must have RPKM ≥ 2 in at least one tissue, 2-fold or greater change between tissues, and P ≤ 0.05. TFs have been grouped by family.

The paper mulberry has developed horizontal, strong lateral and densely tangled fibrous root which can effectively absorb the water and nutrients existing in the topsoil to accommodate the poor soil and harsh environmental conditions. According to our results, a total of 135 TFs belonged to 40 families specifically higher expressed in the root than that in shoot and leaf. It included ARR-B (8), bHLH (15), CO-like (6), G2-like (2), GATA (3), and MYB (8) and so on (see Additional file 2: Figure S1, Additional file 3: Table S5 and Figures 4 and 7).

Investigations on the growth and development of plant roots mainly lie in the top of the regulation of root apical meristem, lateral roots and root hairs growth and development. ARFs promote lateral root growth via an auxin-responsive regulatory network [30] while *NAC1* down-regulate auxin signals for Arabidopsis lateral root development [31]. Auxin targets elongating epidermal cells during the gravitropic response and also regulates cell division in the meristem and stem cell niche [32]. Two *ARF*s and 6 *NAC*s highly expressed in the root (see Additional file 3: Table S5) might be the candidate gene that control the growth of lateral root and root tip in the paper mulberry. In addition, COL3 as a positive regulator of photomorphogenesis can promote lateral root development independently of *COP1* and also function as a day length-sensitive regulator of shoot branching [33]. Six *CO-like*s highly expressed in the root and four of them showed the root-specific expression, which was thought to promote the lateral root development of the paper mulberry.

Genetic analyses suggest that *AtMYC2* belongs to bHLH family and is a common TF involving in light, ABA, and JA signaling pathways. It acts as a negative regulator of blue light-mediated photomorphogenic growth and blue and far-red-light–regulated gene expression, meanwhile it functions as a positive regulator of lateral root formation [34]. *MYC3*, another bHLH TF, directly interactes with JAZs via its N-terminal region and regulate JA responses. The transgenic plants with overexpression of *MYC3* exhibit hypersensitivity in JA-inhibitory root elongation and seedling development [35]. A *bHLH* TF, RSL4 is sufficient to promote postmitotic cell growth in Arabidopsis root-hair cells. Loss of RSL4 function resulted in the development of very short root hairs. In contrast, constitutive *RSL4* expression programs constitutive growth and results in the formation of very long root hairs. Hair-cell growth signals, such as auxin and low phosphate availability, modulate hair cell extension by regulating *RSL4* transcript and its protein levels [36]. A total of 15 highly expressed *bHLH*s in the root implied their function in the lateral root formation as well as the root hairs development via the perception of auxin and other circumstance signals in the paper mulberry.

Cross-talk exists among phytohormones signaling pathways. For example, root meristem size and root growth are mediated mainly by the interplay between cytokinin and auxin. Cytokinin activates *ARR-B* TFs which promote the expression of *SHY2* and affects auxin signaling pathway [37]. *ARR10* and *ARR12* have been proved that they are involved in the AHK-dependent signaling pathway that negatively regulates the protoxylem specification in root vascular tissues [38]. Twelve *ARR-B*s highly expressed in the root and 8 of them showed the root-specific expression (see Additional file 3: Table S5) in the paper mulberry. Thus we proposed that those *ARR-B* TFs redundantly played pivotal roles in response to cytokinin and interacted with the auxin signaling pathway in root growth of the paper mulberry.

Ethylene regulates cell division in quiescent center and auxin biosynthesis in columella cells, which is likely to be involved in root meristem maintenance. In the ethylene signaling pathway, the activated EIN2 promotes the activation of *EIN3* and *EIN3-lik*e (*EIL*) TFs, which induces the expression of *ERF* which is another TF implicated in the activation of a subset of ethylene response genes [32]. Thus, 4 *EIL*s expressed in the root might induce the expression of *ERFs* which expression level was higher than leaf or shoot, and then activated a series of downstream genes to regulate the root meristem maintenance.

Alfin-like TF is involved in the root growth and controls the target genes which are crucial for the root hair elongation [39]. Two *Alfin-like*s showed the highly expressed in the root, suggesting their function in the root hair growth of the paper mulberry.

Although *G2-like* (GOLDEN2-LIKE) TFs are required for chloroplast development and have been reported to co-regulate and synchronize the expression of a suite of nuclear photosynthetic genes and thus act to optimize photosynthetic capacity in varying environmental and developmental conditions, two *G2-like*s were root-specifically expressed and other six *G2-like*s also showed the higher expression characteristic, which implied that those *G2-like*s involved in controlling of root growth and suggested that their functional diverse in the regulation of plant development.

Besides, many other TFs, such as *GATA*s, *GRAS*s, *HSF*s, *NF-YB*s, *Trihelix* and *ZF-HD* also showed the root-specific expression or highly expressed in root than in leaf or shoot, suggesting their complicated cross-talk in the regulation of root growth in the paper mulberry.

**Figure 8:**
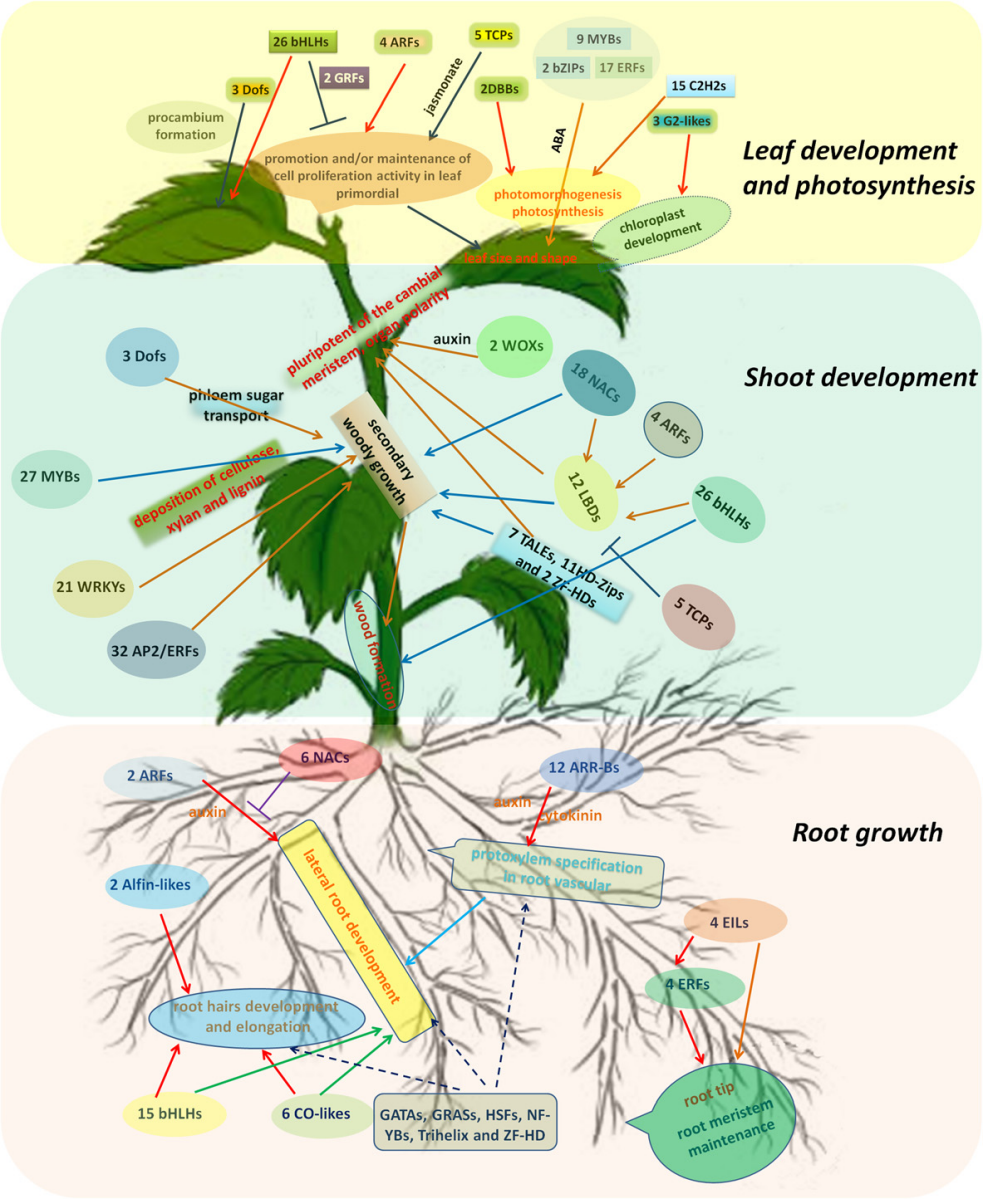
**The proposed TFs involved in tissues development and growth of the paper mulberry.** The arrow lines stand for the effect of activation. The “T” lines stand for the effects of inhibition. The dotted lines stand for the unknown effects.

Many root-specific expressed and highly expressed TFs belonged to the *ARR-B*, *ARF*, *NAC* and *bHLH* family, which might play key roles in the lateral root development under the interaction with kinds of plant hormones and other TFs, though lest specifically expressed TFs were identified in the root compared with shoot and leaf. This might account for the developed lateral root and without an obvious taproot in the paper mulberry (Figure 8).

#### Shoot development

The shoot of the paper mulberry is the tissue of elongation growth and shoot apical meristem, lateral meristem development. Being rich in branches, the shoot of the paper mulberry grows quickly, especially during secondary growth. In our study, a total of 296 TFs belonged to 42 families specifically higher expressed in the shoot than that in root and leaf. It included bHLH (26), Dof (15), ERF (26), LBD (12) and WOX (2) and so on (see Additional file 3: Table S5 and Figure 6). These TFs might govern the complex networks of transcriptional regulation during the shoot development in the paper mulberry.

Indeed, transcriptional profiling indicates that many genes encoding TFs are expressed preferentially during wood formation in various plant species and specific TFs might regulate their expression in a coordinated fashion [14]. Many TFs have been reported to play roles in vascular and xylem development, maintenance of procambium in stem [40,41]. For example, a total of 439 TFs are differentially expressed during shoot development in *Populus*, including *MYB*s, *NAC*s, and *ERF*s [13].

Diverse MYB TFs may participate in the development of vascular tissues and the tension wood response. *PttMYB21a* expression is much higher in secondary cell wall formation zone of xylem and phloem fibers than in other developmental zones. Transgenic expression lines show the reduced growth and had fewer internodes compared to the wild-type, suggesting that *PttMYB21a* might function as a transcriptional repressor in shoot growth [42]. Overexpression of *PtrMYB3* or *PtrMYB20* increases deposition of cellulose, xylan and lignin in *Arabidopsis*. Besides, expression of *PtrMYB3* and *PtrMYB20* is directly activated by PtrWND2, a NAC TF which preferentially expressed in developing wood [40]. Out of the expressed 69 *MYB*s in shoot of the paper mulberry, more than 47.8% showed the shoot growth associated expression patterns. Similarly to *MYB*s, 18 *NAC*s were higher expressed in shoot (Figure 7). These data together suggest that the enriched *NAC* and *MYB* TFs in the shoot implied their function in regulating wood formation in the paper mulberry.

Although WRKY TFs are mainly implicated in regulating defense signal [43], they have also been identified to be highly expressed in Arabidopsis stem of secondary growth and xylem tissue [44]. There were 21 WRKY members that were highly expressed in the shoot of the paper mulberry (Figure 6). The functional study of those WRKYs would help to expand knowledge of the diversity of WRKY developmental functions in this tree.

There were 32 *AP2/ERF*s which was the largest TF family that highly expressed in the shoot of the paper mulberry (see Additional file 5: Table S2). AP2/ERF family members are known to be involved in integration of jasmonic acid and ethylene signals in plant defense [8,45,46], but also have members that affect cell expansion, proliferation and differentiation pathways in Arabidopsis [47,48]. It has also been identified in aspen as differentially expressed at phloem localized in secondary tissue [49].

The role of Dof TFs, a group of plant-specific TFs, recently emerged as part of the transcriptional regulatory networks acting on the formation and functioning of the vascular tissues. Some of the Dof TF genes (*AtDof2.4*, *AtDof5.8* and *AtDof5.6*/*HCA2*) are reported to be expressed specifically in cells at an early stage of vascular tissue development [9]. Besides, *AtDof* TFs also potentially control the phloem sugar transport. Therefore, 3 *Dof*s high expressed in the shoot implied their important and diverse functions in the vascular tissue development of the the paper mulberry shoot.

The *TALE*, *HD-Zip*, *WOX* and *ZF-HD* homeodomain containing TFs have been associated with processes related to meristem function, organ polarity, and vascular development in several species [13]. There were TALE (4), HD-Zip (8), WOX (2) and ZF-HD (2) showed the shoot-specific expression in the paper mulberry.

The maintenance of the pluripotent identity of the cambium is crucial for the continuous meristem activity. Current evidence indicates that a similar molecular mechanism regulating shoot apical meristem (SAM) and root apical meristem (RAM) is likely applicable in cambial meristems. The WOX TFs function have been identified in SAM and RAM by a dynamic feedback loop involving the CLAVATA3 (CLV3) peptide ligand and the CLV1 receptor in SAM [50]. Two *WOX*s (T2-25818 and T2-23235) exhibited shoot-specific expression, which suggested their key roles in the maintenance of the pluripotent of the cambial meristem during the shoot development of the paper mulberry.

There were 12 *LBD*s highly expressed in shoot than that in leaf and root. LBD family is plant-specific TF and has been implicated in plant development. Two members of the *Arabidopsis* LBD family, *AS2-LIKE19 (ASL19)/LBD30* and *ASL20/LBD18* were expressed in immature tracheary elements (TEs), and the expression was dependent on *VND6* and *VND7*, which are *NAC* TFs required for TE differentiation. *ASL20* appears to be involved in a positive feedback loop for *VND7* expression that regulates TE differentiation-related gene [51]. Dominant-negative suppression of *PtaLBD1* via translational fusion with the repressor SRDX domain caused decreased diameter growth and highly irregular phloem development. In wild-type plants, *LBD1* was most highly expressed in the phloem and cambial zone. These results suggested that a broader regulatory role of LBD during secondary woody growth in Poplar [52]. Four *LBD* genes downstream of ARFs, *LBD16*, *LBD17*, *LBD18* and *LBD29*, are rapidly and dramatically induced by callus-inducing medium, LBD as key regulators in the callus induction process, thereby establishing a molecular link between auxin signaling and the plant regeneration program [53].

In addition, *bHLH048* regulates the function of LOB (namely LBD TF) at lateral organ boundaries [54]. However, TCP TFs play a pivotal role in the control of morphogenesis of shoot organs by negatively regulating the expression of boundary specific genes, including *LBD*s [55]. Five *TC*Ps highly expressed in shoot of the paper mulberry, which might negatively regulated the expression of *LBD*s in shoot and this was consistent with that, except T7-29380, the all of shoot-specific *LBD*s RPKM was relatively lower.

Besides, *ARF* (4), *bHLH* (26) and *NAC* (18) were shoot-specific expression, which might directly or indirectly involve in the shoot development via the regulation of LBD in the paper mulberry. It seemed that LBD TFs were the center link in the regulation of shoot development in the paper mulberry (Figure 8).

#### Leaf development and photosynthesis

Leaves are photosynthetic organs. Thus, the shapes and sizes of leaves are very important factors influencing the success of plants. In our study, a total of 196 TFs belonged to 33 families were specifically higher expressed in the leaf than shoot and root. It included *bHLH*s (26), *C2H2* (15), *ERF* (17) and *TCP* (5) and so on (see Additional file 3: Table S5 and Figure 7). These TFs might have the important regulated effect on the photosynthesis and leaf development in the paper mulberry.

*Atgrf5* mutants exhibit narrow-leaf phenotypes due to decreases in cell number. Conversely, cell proliferation in leaf primordia is enhanced and leaves grow larger than normal when *AtGRF5* is overexpressed. These results suggest that *AtGRF5* is required for the development of appropriate leaf size and shape through the promotion and/or maintenance of cell proliferation activity in leaf primordial [56]. The *SPT* gene, encoding a bHLH TF, functions as a repressor of leaf growth and acts independently from another set of cell proliferation dependent organ size regulators, *AN3* and *AtGRF5* [7]. So, 4 *ARF*s and 26 *bHLH*s might play the major roles in the development of appropriate leaf size and shape of the paper mulberry.

A loss-of function of the *ANT* gene, a member of the AP2/ERF family, typically results in small leaves with fewer cells of larger volume as compared with wild-type cells. In contrast, *ANT* overexpression in petals causes an increase in cell number without a change in cell size [57]. A total of 17 *ERF*s highly expressed in the leaf might play different roles from the shoot-specific expressed members, involving the leaf development of the paper mulberry.

BBX proteins are key factors in regulatory networks controlling growth and developmental processes that include seedling photomorphogenesis, photoperiodic regulation of flowering, shade avoidance, and responses to biotic and abiotic stresses [58]. Their functions are not totally redundant, as judged by the fact that some DBBs were apparently implicated in light signal transduction in a negative manner, whereas others were implicated in a positive manner with regard to light-induced inhibition of elongation of hypocotyls [59]. For instance, BBX25 and BBX24 function as transcriptional co-repressors forming inactive heterodimers with HY5 (a bZIP TF) down regulating BBX22 expression for the fine-tuning of light-mediated seedling development [60]. Therefore, two leaf-specific expressed *DBB*s were considered to involve in the photomorphogenesis of the paper mulberry.

*AtDof* control the procambium formation during leaf development [9] and its homologous in the paper mulberry may function in the formation of procambium. TCP3, a model of CIN-like TCPs of Arabidopsis, plays important roles in the signaling pathways that generate different leaf forms without having any lethal effects on shoots by directly activating the expression of *miR164*, *AS1*, *IAA3/SHY2*, and *SAUR* [61]. In addition, analysis of *tcp9* and *tcp20* mutants exhibits an antagonistic function of TCP9 and TCP20 proteins in the control of leaf development via the jasmonate signaling pathway [62]. Recent study reveal that TIE1, as a major modulator of TCP activities during leaf development, may interact with both TCPs and TPL/TPRs to form transcriptional repressor complexes to repress the expression of TCP target genes, thus preventing the cells in young leaves from undergoing differentiation. In mature leaves, *TIE1* expression is decreased and the activities of TCP proteins may not be inhibited by TIE1. Therefore, the downstream genes of TCPs are activated to promote cell differentiation. Overexpression of *TIE1* leads to hyponastic and serrated leaves, whereas disruption of *TIE1* causes epinastic leaves [63]. So, 5 highly expressed *TCP*s in the leaf might involve in the regulation of leaf forms of the paper mulberry (Figure 8).

In several land plants, G2-like TFs are required for chloroplast development. Double mutants of *glk1* and *glk2* Arabidopsis accumulate abnormal levels of chlorophyll precursors and constitutive *GLK* gene expression leads to increased accumulation of transcripts for antenna proteins and chlorophyll biosynthetic enzymes. *GLK* genes help to co-regulate and synchronize the expression of a suite of nuclear photosynthetic genes and thus act to optimize photosynthetic capacity in varying environmental and developmental conditions [64]. Three *G2-like*s were leaf-specific expression and its three paralogous were shoot-specific expression which suggested their different functions during chloroplast development in the leaf and shoot of the paper mulberry. However, there were also 5 *G2-like*s that exhibited the root-specific expression, which implied their different roles in the root growth of the paper mulberry and the essentiality of further study.

Collectively, our results indicated that leaf-specific expression TFs were focus on the families which played significant roles in the leaf development, such as AP2/EREBP, bHLH, GRFs, TCPs, as well as the families functioned in photomorphogenesis and photosynthesis, like DBBs, G2-like and so on. Together with C2H2, MYB, NAC and WRKY, all of these leaf-specific expressed TFs corporately regulated the leaf development, photosynthesis and carbohydrate metabolism in the paper mulberry (Figure 8).

## Conclusion

Our study is the comprehensive transcriptome-wide identification of TFs in the paper mulberry without genome information as reference. More importantly, a large numbers of TFs regulated the lateral organ growth are tissue-specific expression, which may contribute for the developed lateral roots, more branches and rapid growth of the paper mulberry. Of course, the more specific functional differentiation of those TFs need further study. Transcriptomics-based identification of these TFs, particularly the tissue-specific expression TFs genes, provides important information for understanding the development and transcriptional regulation of the paper mulberry and leads to potential applications in the development of genetically modified with the paper mulberry.

## Methods

### Plant material and RNA extraction

Plantlets were cultured on MS culture media in an artificial climatic chamber kept at 26°C and a 14/10 h photoperiod (day/night). In this study, a mixed sampling strategy was adopted to eliminate differences between individuals.

Total RNA was isolated with TRIzol® Reagent (Life technologies, Shanghai, China) from each sample according to the manufacturer’s instruction. It was treated with RNase-free DNase I (Takara, Dalian, China) to remove the residual DNA. RNA quality and purity were assessed with OD260/230 ratio and RNA integrity number (RIN) by using the NanoDrop 2000 (Thermo Fisher, Waltham, USA) and the Agilent 2100 Bioanalyzer (Agilent Technologies, Santa Clara, USA), respectively.

### Sequence retrieval, identification, classification and expression analysis of TFs

Raw sequence data were generated by Illumina pipeline and have been available in NCBI’s Short Read Archive (SRA) database (http://www.ncbi.nlm.nih.gov/Traces/sra/sra.cgi) under accession number SRP029966. All of the Illumina reads generated from different cDNA libraries were *de novo* assembled with Trinity program to form the global transcriptome of the paper mulberry [22]. For the functional annotation, unigenes were firstly aligned by Blastx to protein databases nr (E ≤ 1e-5), retrieving proteins with the highest sequence similarity to the given unigenes along with their functional annotations. After getting annotation result for every unigene, all of the TFs of the paper mulberry were identified and classified into different families based on their DNA-binding domains and other conserved motif [18]. Based on the alignment results of orthologous in the NCBI using the Blastx tool, the TFs would be determined whether they contained the complete ORF. In addition, all of the TF families’ abbreviations presented in this paper were referenced to Plant TFDB3.0 [18].

For gene expression analysis, the expression level of each TF in each sample was calculated by quantifying the number of Illumina reads that mapped to transcriptome of the paper mulberry. The raw gene expression counts were normalized using the RPKM method (Reads per kb per million reads).

### Phylogenetic analysis of TF families

TFs with the completed ORF of CAMTA, VOZ and Whirly families were used to do phylogenetic analysis. The TF family protein sequences of *Amborella trichopoda*, *Arabidopsis thaliana*, *Brachypodium distachyon*, *Cannabis sativa*, *Citrullus lanatus*, *Fragaria vesca*, *Linum usitatissimum*, *Nelumbo nucifera*, *Oryza sativa* subsp. *Japonica*, *Prunus persica*, *Sorghum bicolor* and *Vitis vinifera* were downloaded from Plant Transcription Factor Database (http://planttfdb.cbi.pku.edu.cn/index.php). The TFs of *Morus notabilis* were searched from the Morus Genome Database (http://morus.swu.edu.cn/morusdb/). The information of these TFs was listed in Additional file 6: Table S1. Phylogenetic and molecular evolutionary analyses were conducted using MEGA (version 5.0) with pairwise distance and Neighbor- Joining algorithm. The evolutionary distances were computed using the p-distance method and were used to estimate the number of amino acid substitutions per site. The reliability of each tree was established by conducting 1500 bootstrap sampling steps.

### Identification of differentially expressed TFs

For screening of differentially expressed TFs, p value corresponds to differentially expressed genes (DEGs) was obtained via a general *Chi* squared test that was performed by using IDEG6 (http://telethon.bio.unipd.it/bioinfo/IDEG6/). The threshold of p value in multiple tests was checked through manipulating the false discovery rate (FDR) value. The TF with ratio of RPKM between samples of more than 2 (Fold change ≥2) and the FDR ≤ 0.01 was taken as the significantly difference expressed TF. The MeV (Multiexperiment Viewer, v4.9) was used to make the heat map and expressing pattern classification.

### Validation by qPCR

Real time PCR was adopted to validate the DEGs identified in analysis of the RNA-seq data. Ten TFs were chosen for verification (see Additional file 5: Table S2). RNA used for validation was the same as that isolated for RNA-seq. First-strand cDNA for each sample was made from 1 μg of total RNA using SuperScript II reverse transcriptase (Takara, Dalian, China) following the manufacturer’s recommendations and diluted 3 times before use in PCR. Gene-specific primers based on the selected considerate unigenes were subsequently designed using the Primer premier 5.0 program and are list in Additional file 5: Table S2. Real-Time PCR reaction condition and volume was performed as described by in our former study [22]. Relative transcript levels for each sample were obtained using the comparative cycle threshold method using the cycle threshold value of the *GAPDH* gene for each sample as a standard.

## Additional files

Additional file 1: Table S4. The expression profile of all the TFs in paper mulberry. Additional file 2: Figure S1. The differentially expressed TFs distributed in every family. Additional file 3: Table S5. The expression profile of the TFs with differential expression among root, shoot and leaf. Additional file 4: Table S6. Differentially expressed TF families with complete ORF in paper mulberry. Additional file 5: Table S2. The primers designed for the selected TFs and used for qPCR. Additional file 6: Table S1. The accession number and the sequence information of the TFs from the selected species. TFs of CAMTA, VOZ and Whirly families of *Amborella trichopoda*, *Arabidopsis thaliana*, *Brachypodium distachyon*, *Cannabis sativa*, *Citrullus lanatus*, *Fragaria vesca*, *Linum usitatissimum*, *Nelumbo nucifera*, *Oryza sativa* subsp. *Japonica*, *Prunus persica*, *Sorghum bicolor* and *Vitis vinifera* were downloaded from Plant Transcription Factor Database (http://planttfdb.cbi.pku.edu.cn/index.php). The TFs of *Morus notabilis* were searched from the Morus Genome Database (http://morus.swu.edu.cn/morusdb/). All the TFs were re-named for the convenience of phylogenetic analysis.

## References

[1] Saito K, Linquist B, Keobualapha B, Shiraiwa T, Horie T (2009). *Broussonetia papyrifera* (paper mulberry): its growth, yield and potential as a fallow crop in slash-and-burn upland rice system of northern Laos. Agrofor Syst.

[2] Zhai X, Zeng H, Liu Y, Liu F (2012). Change of nutrients and shape of *Broussonetia papyrifera* leaves from different clones. J Northeast Forestry Univ.

[3] Sun J, Peng X, Fan W, Tang M, Liu J, Shen S (2013). Functional analysis of *BpDREB2* gene involved in salt and drought response from a woody plant *Broussonetia papyrifera*. Gene.

[4] Yan J, Wu P, Du H, Zhang Q (2011). First report of black spot caused by Colletotrichum gloeosporioides on paper Mulberry in China. Plant Dis.

[5] Wang J, Liu J, Peng X, Ni Z, Wang G, Shen S (2014). Applied hybrid paper mulberry in ecological virescence of the coastal saline. Tianjin Agric Sci.

[6] Liu Z, Fan W, Shen S (2009). SRAP marker in *Broussonetia papyrifera*. Scientia Silvae Sinicae.

[7] Ichihashi Y, Horiguchi G, Gleissberg S, Tsukaya H (2010). The bHLH transcription factor SPATULA controls final leaf size in *Arabidopsis thaliana*. Plant Cell Physiol.

[8] Peng X, Zhang L, Zhang L, Liu Z, Cheng L, Yang Y, Shen S, Chen S, Liu G (2013). The transcriptional factor LcDREB2 cooperates with LcSAMDC2 to contribute to salt tolerance in *Leymus chinensis*. Plant Cell Tissue Organ Cult.

[9] Le Hir R, Bellini C (2013). The plant-specific Dof transcription factors family: new players involved in vascular system development and functioning in Arabidopsis. Front Plant Sci.

[10] Zhu J, Verslues PE, Zheng X, Lee BH, Zhan X, Manabe Y, Sokolchik I, Zhu Y, Dong C-H, Zhu J-K (2005). *HOS10* encodes an R2R3-type MYB transcription factor essential for cold acclimation in plants. Proc Natl Acad Sci U S A.

[11] He XJ, Mu RL, Cao WH, Zhang ZG, Zhang JS, Chen SY (2005). AtNAC2, a transcription factor downstream of ethylene and auxin signaling pathways, is involved in salt stress response and lateral root development. Plant J.

[12] Berri S, Abbruscato P, Faivre-Rampant O, Brasileiro A, Fumasoni I, Satoh K, Kikuchi S, Mizzi L, Morandini P, Pè M (2009). Characterization of WRKY co-regulatory networks in rice and Arabidopsis. BMC Plant Biol.

[13] Dharmawardhana P, Brunner AM, Strauss SH (2010). Genome-wide transcriptome analysis of the transition from primary to secondary stem development in *Populus trichocarpa*. BMC Genomics.

[14] Demura T, Fukuda H (2007). Transcriptional regulation in wood formation. Trends Plant Sci.

[15] Hu R, Qi G, Kong Y, Kong D, Gao Q, Zhou G (2010). Comprehensive analysis of NAC domain transcription factor gene family in *Populus trichocarpa*. BMC Plant Biol.

[16] Hedman H, Zhu T, von Arnold S, Sohlberg JJ (2013). Analysis of the WUSCHEL-RELATED HOMEOBOX gene family in the conifer *picea abies* reveals extensive conservation as well as dynamic patterns. BMC Plant Biol.

[17] Du J, Groover A (2010). Transcriptional regulation of secondary growth and wood formation. J Integr Plant Biol.

[18] Jin J, Zhang H, Kong L, Gao G, Luo J (2013). PlantTFDB 3.0: a portal for the functional and evolutionary study of plant transcription factors. Nucleic Acids Res.

[19] Pérez-Rodríguez P, Riaño-Pachón DM, Corrêa LGG, Rensing SA, Kersten B, Mueller-Roeber B (2010). PlnTFDB: updated content and new features of the plant transcription factor database. Nucleic Acids Res.

[20] Yilmaz A, Mejia-Guerra MK, Kurz K, Liang X, Welch L, Grotewold E (2011). AGRIS: the Arabidopsis gene regulatory information server, an update. Nucleic Acids Res.

[21] Tuskan GA, Difazio S, Jansson S, Bohlmann J, Grigoriev I, Hellsten U, Putnam N, Ralph S, Rombauts S, Salamov A (2006). The genome of black cottonwood, *Populus trichocarpa* (Torr. & Gray). Science.

[22] Peng X, Teng L, Wang X, Wang Y, Shihua S (2014). De Novo assembly of expressed transcripts and global transcriptomic analysis from seedlings of the Paper Mulberry (*Broussonetia kazinoki* x *Broussonetia papyifera*). PLoS One.

[23] Finkler A, Ashery-Padan R, Fromm H (2007). CAMTAs: calmodulin-binding transcription activators from plants to human. FEBS Lett.

[24] Yang T, Peng H, Whitaker BD, Conway WS (2012). Characterization of a calcium/calmodulin-regulated SR/CAMTA gene family during tomato fruit development and ripening. BMC Plant Biol.

[25] Albert VA, Barbazuk WB, Der JP, Leebens-Mack J, Ma H, Palmer JD, Rounsley S, Sankoff D, Schuster SC, Soltis DE (2013). The Amborella genome and the evolution of flowering plants. Science.

[26] Desveaux D, Maréchal A, Brisson N (2005). Whirly transcription factors: defense gene regulation and beyond. Trends Plant Sci.

[27] Nakai Y, Nakahira Y, Sumida H, Takebayashi K, Nagasawa Y, Yamasaki K, Akiyama M, Ohme‐Takagi M, Fujiwara S, Shiina T (2013). Vascular plant one-zinc-finger protein 1/2 transcription factors regulate abiotic and biotic stress responses in Arabidopsis. Plant J.

[28] McPartland JM, Guy GW (2004). The evolution of Cannabis and coevolution with the cannabinoid receptor-a hypothesis. Med Use Cannabis Cannabinoids.

[29] Jiang Y, Zeng B, Zhao H, Zhang M, Xie S, Lai J (2012). Genome‐wide transcription factor gene prediction and their expressional tissue‐specificities in MaizeF. J Integr Plant Biol.

[30] Marin E, Jouannet V, Herz A, Lokerse AS, Weijers D, Vaucheret H, Nussaume L, Crespi MD, Maizel A (2010). miR390, Arabidopsis TAS3 tasiRNAs, and their AUXIN RESPONSE FACTOR targets define an autoregulatory network quantitatively regulating lateral root growth. Plant Cell.

[31] Guo H-S, Xie Q, Fei J-F, Chua N-H (2005). MicroRNA directs mRNA cleavage of the transcription factor NAC1 to downregulate auxin signals for Arabidopsis lateral root development. Plant Cell.

[32] Ubeda-Tomás S, Beemster GT, Bennett MJ (2012). Hormonal regulation of root growth: integrating local activities into global behaviour. Trends Plant Sci.

[33] Datta S, Hettiarachchi G, Deng X-W, Holm M (2006). Arabidopsis CONSTANS-LIKE3 is a positive regulator of red light signaling and root growth. Plant Cell.

[34] Yadav V, Mallappa C, Gangappa SN, Bhatia S, Chattopadhyay S (2005). A basic helix-loop-helix transcription factor in Arabidopsis, MYC2, acts as a repressor of blue light–mediated photomorphogenic growth. Plant Cell.

[35] Cheng Z, Sun L, Qi T, Zhang B, Peng W, Liu Y, Xie D (2011). The bHLH transcription factor MYC3 interacts with the jasmonate ZIM-domain proteins to mediate jasmonate response in Arabidopsis. Mol Plant.

[36] Yi K, Menand B, Bell E, Dolan L (2010). A basic helix-loop-helix transcription factor controls cell growth and size in root hairs. Nat Genet.

[37] Muraro D, Byrne H, King J, Voß U, Kieber J, Bennett M (2011). The influence of cytokinin–auxin cross-regulation on cell-fate determination in *Arabidopsis thaliana* root development. J Theor Biol.

[38] Yokoyama A, Yamashino T, Amano Y-I, Tajima Y, Imamura A, Sakakibara H, Mizuno T (2007). Type-B ARR transcription factors, ARR10 and ARR12, are implicated in cytokinin-mediated regulation of protoxylem differentiation in roots of *Arabidopsis thaliana*. Plant Cell Physiol.

[39] Chandrika NNP, Sundaravelpandian K, Yu SM, Schmidt W (2013). ALFIN-LIKE 6 is involved in root hair elongation during phosphate deficiency in Arabidopsis. New Phytol.

[40] Zhang J, Elo A, Helariutta Y (2011). Arabidopsis as a model for wood formation. Curr Opin Biotechnol.

[41] Plomion C, Leprovost G, Stokes A (2001). Wood formation in trees. Plant Physiol.

[42] Karpinska B, Karlsson M, Srivastava M, Stenberg A, Schrader J, Sterky F, Bhalerao R, Wingsle G (2004). MYB transcription factors are differentially expressed and regulated during secondary vascular tissue development in hybrid aspen. Plant Mol Biol.

[43] He H, Dong Q, Shao Y, Jiang H, Zhu S, Cheng B, Xiang Y (2012). Genome-wide survey and characterization of the *WRKY* gene family in *Populus trichocarpa*. Plant Cell Rep.

[44] Zhao C, Craig JC, Petzold HE, Dickerman AW, Beers EP (2005). The xylem and phloem transcriptomes from secondary tissues of the Arabidopsis root-hypocotyl. Plant Physiol.

[45] Xianjun P, Xingyong M, Weihong F, Man S, Liqin C, Alam I, Lee B-H, Dongmei Q, Shihua S, Gongshe L (2011). Improved drought and salt tolerance of Arabidopsis thaliana by transgenic expression of a novel DREB gene from Leymus chinensis. Plant Cell Rep.

[46] Mizoi J, Shinozaki K, Yamaguchi-Shinozaki K (2012). AP2/ERF family transcription factors in plant abiotic stress responses. Biochim Biophys Acta.

[47] Marsch-Martinez N, Greco R, Becker JD, Dixit S, Bergervoet JH, Karaba A, de Folter S, Pereira A (2006). BOLITA, an Arabidopsis AP2/ERF-like transcription factor that affects cell expansion and proliferation/differentiation pathways. Plant Mol Biol.

[48] Boutilier K, Offringa R, Sharma VK, Kieft H, Ouellet T, Zhang L, Hattori J, Liu C-M, van Lammeren AA, Miki BL (2002). Ectopic expression of *BABY BOOM* triggers a conversion from vegetative to embryonic growth. Plant Cell.

[49] Van Raemdonck D, Pesquet E, Cloquet S, Beeckman H, Boerjan W, Goffner D, El Jaziri M, Baucher M (2005). Molecular changes associated with the setting up of secondary growth in aspen. J Exp Bot.

[50] Van der Graaff E, Laux T, Rensing SA (2009). The WUS homeobox-containing (WOX) protein family. Genome Biol.

[51] Soyano T, Thitamadee S, Machida Y, Chua N-H (2008). Asymmetric leaves2-like19 lateral organ boundaries domain30 and Asl20/Lbd18 regulate tracheary element differentiation in Arabidopsis. Plant Cell.

[52] Yordanov YS, Regan S, Busov V (2010). Members of the LATERAL ORGAN BOUNDARIES DOMAIN transcription factor family are involved in the regulation of secondary growth in Populus. Plant Cell.

[53] Fan M, Xu C, Xu K, Hu Y (2012). LATERAL ORGAN BOUNDARIES DOMAIN transcription factors direct callus formation in Arabidopsis regeneration. Cell Res.

[54] Husbands A, Bell EM, Shuai B, Smith HM, Springer PS (2007). LATERAL ORGAN BOUNDARIES defines a new family of DNA-binding transcription factors and can interact with specific bHLH proteins. Nucleic Acids Res.

[55] Koyama T, Furutani M, Tasaka M, Ohme-Takagi M (2007). TCP transcription factors control the morphology of shoot lateral organs via negative regulation of the expression of boundary-specific genes in Arabidopsis. Plant Cell.

[56] Horiguchi G, Kim GT, Tsukaya H (2005). The transcription factor AtGRF5 and the transcription coactivator AN3 regulate cell proliferation in leaf primordia of *Arabidopsis thaliana*. Plant J.

[57] Mizukami Y, Fischer RL (2000). Plant organ size control: AINTEGUMENTA regulates growth and cell numbers during organogenesis. Proc Natl Acad Sci.

[58] Gangappa SN, Botto JF: **The BBX family of plant transcription factors.** *Trends Plant Sci* 2014. doi:10.1016/j.tplants.2014.01.010. 10.1016/j.tplants.2014.01.010 24582145

[59] Kumagai T, Ito S, Nakamichi N, Niwa Y, Murakami M, Yamashino T, Mizuno T (2008). The common function of a novel subfamily of B-Box zinc finger proteins with reference to circadian-associated events in *Arabidopsis thaliana*. Biosci Biotechnol Biochem.

[60] Gangappa SN, Crocco CD, Johansson H, Datta S, Hettiarachchi C, Holm M, Botto JF (2013). The Arabidopsis B-box protein BBX25 interacts with HY5, negatively regulating *BBX22* expression to suppress seedling photomorphogenesis. Plant Cell.

[61] Koyama T, Mitsuda N, Seki M, Shinozaki K, Ohme-Takagi M (2010). TCP transcription factors regulate the activities of ASYMMETRIC LEAVES1 and miR164, as well as the auxin response, during differentiation of leaves in Arabidopsis. Plant Cell.

[62] Danisman S, Van der Wal F, Dhondt S, Waites R, de Folter S, Bimbo A, van Dijk AD, Muino JM, Cutri L, Dornelas MC (2012). Arabidopsis class I and class II TCP transcription factors regulate jasmonic acid metabolism and leaf development antagonistically. Plant Physiol.

[63] Tao Q, Guo D, Wei B, Zhang F, Pang C, Jiang H, Zhang J, Wei T, Gu H, Qu L-J (2013). The TIE1 transcriptional repressor links TCP transcription factors with TOPLESS/TOPLESS-RELATED corepressors and modulates leaf development in Arabidopsis. Plant Cell Online.

[64] Waters MT, Wang P, Korkaric M, Capper RG, Saunders NJ, Langdale JA (2009). GLK transcription factors coordinate expression of the photosynthetic apparatus in Arabidopsis. Plant Cell.

