# Supplementary figures and images for: Global transcriptomics identification and analysis of transcriptional factors in different tissues of the paper mulberry
Source: BMC Plant Biol. 2014 Aug 20;14:194. doi: 10.1186/s12870-014-0194-6 (PMC4205299; doi:10.1186/s12870-014-0194-6)

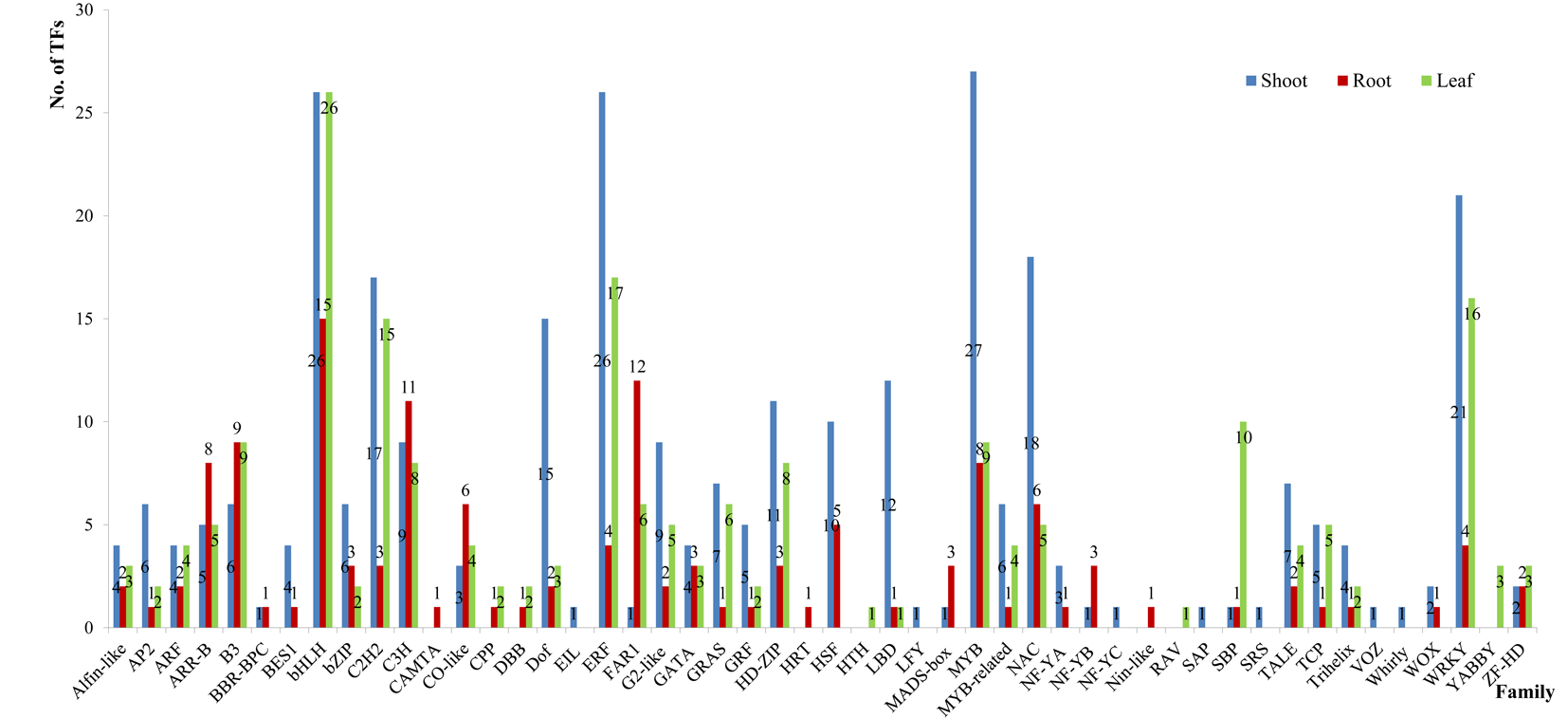

Supplement: Additional file 2: Figure S1. — The differentially expressed TFs distributed in every family. [file 12870_2014_194_MOESM2_ESM.tiff]
