# Supplementary material for: Global transcriptomics identification and analysis of transcriptional factors in different tissues of the paper mulberry
Source: BMC Plant Biol. 2014 Aug 20;14:194. doi: 10.1186/s12870-014-0194-6 (PMC4205299; doi:10.1186/s12870-014-0194-6)
Supplement: Additional file 5: Table S2. — The primers designed for the selected TFs and used for qPCR. [file 12870_2014_194_MOESM5_ESM.docx]

| TF family | ID of selected TF | Name of Primer | Primer Sequence 5' to 3' |
| --- | --- | --- | --- |
| bHLH | T3-16626 | Bp1-F | CCAGAATCTTCCGAAACAAATTATACC |
|  |  | Bp1-R | CTAGACCTGCAATCTCATCTTTCAGC |
| C2H2 | T6-27415 | Bp2-F | AGCTTAACATGGCTTCCTCTAGTGC |
|  |  | Bp2-R | TGTTTCTCACGATCTCTCCAACG |
| C3H | T7-31374 | Bp3-F | TGAGAACGCCAGAAGAAGAGACC |
|  |  | Bp3-R | GCTAACTTTGCTCTGCCACAACC |
| CO-like | T4-26422 | Bp4-F | GAACTCTTTCGTGCCTGCTGG |
|  |  | Bp4-R | TCAAGTAATTTTTCTTTTCCCTCCTGC |
| Dof | T2-27365 | Bp5-F | CAACCCAACACTGCGATATGC |
|  |  | Bp5-R | CCCATAATCTGATGATCCCCACC |
| ERF | T2-19028 | Bp6-F | ACTCTCGCTCGATCTCAACCTACC |
|  |  | Bp6-R | AACCTCTCTCTCTCACATTCACAGC |
| GATA | T3-23795 | Bp7-F | TTTCCCTCAAGACGAGAAAGACG |
|  |  | Bp7-R | AGCAGATGCTAAGGAACAAGAGATCC |
| HD-ZIP | T2-28861 | Bp8-F | GTACTCCTGAAGCACAGACACTTGC |
|  |  | Bp8-R | AAGTAAAACCCTGTTGCATTATCTGC |
| HSF | T4-23436 | Bp9-F | ACATGCCATTTCAGTCCGACG |
|  |  | Bp9-R | ATCTCATTGCCACCAGTAGACATAGC |
| TALE | T6-28724 | Bp10-F | GGTGAACTCGTTTGATCTAGTGATGG |
|  |  | Bp10-R | TTGAAACCTGATTCCTCGATAGACC |
